# Aspirin for the primary prevention of cardiovascular disease in the elderly

**DOI:** 10.1136/bmjebm-2018-111138

**Published:** 2019-02-06

**Authors:** Jack W O’Sullivan

**Affiliations:** 1 Division of Cardiology, Department of Medicine, Stanford University, Stanford, California, USA; 2 Centre for Evidence-Based Medicine, University of Oxford, Oxford, Oxfordshire, UK; 3 Stanford University, Stanford, California, USA

**Keywords:** cardiac epidemiology, cardiology, primary care, general medicine

*The tension between the benefits and harms of anticoagulation is finely balanced. Prophylactic aspirin in healthy, elderly patients provides no benefit and causes harm; clinicians should not use it for primary prevention in otherwise well patients aged over 70.*

As the world’s population ages, health in later life has become a public health priority. The prevention of disease is critical to these aims. As a chief cause of morbidity in the elderly,^1^ the prevention of cardiovascular disease is of particular focus. 

The use of aspirin unequivocally benefits patients who have already suffered a cardiovascular event.^2 3^ However, its role in primary prevention is much more contentious.^4–6^ The Aspirin in Reducing Events in the Elderly (ASPREE) trial^7^ set out to address the uncertainty surrounding the prophylactic use of aspirin in healthy, elderly patients.

The ASPREE investigators randomised almost 20 000 people to receive 100 mg aspirin or placebo. Included participants were 70 years or older (or 65 if Hispanic or African–American) and had no history of cardiovascular disease, dementia, terminal illness or increased risk of bleeding. The rate of cardiovascular disease and haemorrhage were assessed over approximately 5 years (median follow-up 4.7).

This trial proved that the use of aspirin is harmful to healthy, community-dwelling elderly people. Participants that took aspirin derived no benefit but suffered more haemorrhagic events than those that took placebo. People in the aspirin group had 8.6 major haemorrhage events per 1000 person-years compared with 6.2 events per 1000 person-years in the placebo group (HR, 1.38; 95% CI 1.18 to 1.62). The rate of cardiovascular disease for people in the aspirin group was 10.7 events per 1000 person-years, compared with 11.3 in the placebo group (HR, 0.95; 95% CI 0.83 to 1.08).

There are a few limitations to consider. Most significantly, only around two-thirds of the participants were still taking their aspirin by the end of the trial. This could have underestimated the effects of aspirin, but this is likely to have affected both the potential benefits and the harms equally. Furthermore, a small proportion of patients were taking aspirin before the trial started.

This trial has many important implications. Previous studies have provided heterogeneous estimates of the benefit of aspirin for primary prevention,^4^ but pointed to an increased benefit of aspirin in the elderly. The ASPREE trial has shown that elderly patients that take aspirin prophylactically will derive only harm. At this stage, clinical practice guidelines should not recommend the use of aspirin for otherwise well patients aged 70 or older (or 65 if African–American or Hispanic). Many guidelines already advise not to use aspirin for primary prevention, including the National Institute for Health and Clinical Excellence guidelines.^8^ However, other guideline organisations offer more circumspect guidance and acknowledge the previous lack of evidence. For instance, the United States Prevention Task Force currently states: ‘The current evidence is insufficient to assess the balance of benefits and harms of initiating aspirin use for the primary prevention of CVD and CRC in adults aged 70 years or older.’^9^ The ASPREE trial should lead to a change of guidance.

The study authors of the ASPREE trial published two other linked randomised controlled trials investigating the effect of aspirin on all-cause mortality^10^ and disability free-survival.^11^ The results of these studies further support the recommendation not to use aspirin prophylactically in the elderly. Patients that took aspirin had higher rates of all-cause mortality (HR, 1.14; 95% CI 1.01 to 1.29); specifically they had higher rates of cancer-related death (HR, 1.31; 95% CI 1.10 to 1.56). Furthermore, the rate of disability (a composite of death, dementia or persistent physical disability) was no different between those that took aspirin and those that took a placebo (HR, 1.01; 95% CI 0.92 to 1.11; p=0.79).

**EBM verdict**

EBM Verdict on: Effect of Aspirin on Cardiovascular Events and Bleeding in the Healthy Elderly. McNeil JJ et al. N Engl J Med 2018;379:1509–1518. doi: 10.1056/NEJMoa1805819. [Epub 16 Sep 2018].

- In healthy, community-dwelling elderly people aged 70 and older, aspirin does not prevent cardiovascular disease and does increase one’s risk of major haemorrhage. Clinicians should not offer aspirin as primary prevention to otherwise well elderly patients.
